# Platelet indices in stable chronic obstructive pulmonary disease – association with inflammatory markers, comorbidities and therapy

**DOI:** 10.11613/BM.2020.010701

**Published:** 2019-12-15

**Authors:** Iva Hlapčić, Anita Somborac-Bačura, Sanja Popović-Grle, Andrea Vukić Dugac, Dunja Rogić, Ivana Rako, Tihana Žanić Grubišić, Lada Rumora

**Affiliations:** 1 University of Zagreb, Faculty of Pharmacy and Biochemistry, Department of Medical Biochemistry and Haematology, Zagreb, Croatia; 2 University Hospital Centre Zagreb, Clinical Department for Lung Diseases Jordanovac, Zagreb, Croatia; 3 University of Zagreb, School of Medicine, Zagreb, Croatia; 4 University Hospital Centre Zagreb, Clinical Institute of Laboratory Diagnostics, Zagreb, Croatia

**Keywords:** chronic obstructive pulmonary disease, inflammation, blood platelets, therapy

## Abstract

**Introduction:**

Chronic obstructive pulmonary disease (COPD) is a complex inflammatory condition that can affect haemostasis. This study aimed to determine differences in platelet-related parameters between controls and COPD subjects. The hypothesis was that platelet indices are disturbed in COPD patients, and this would be accompanied by increased C-reactive protein (CRP), fibrinogen (Fbg) and white blood cells (WBC). Therefore, platelet count (Plt), platelet-related parameters – mean platelet volume (MPV), platelet distribution width (PDW), plateletcrit (Pct), their ratios (MPV/Plt, MPV/Pct, PDW/Plt, PDW/Pct), platelet to lymphocyte ratio (PLR), Plt index as well as CRP, Fbg and WBC were assessed.

**Materials and methods:**

Study included 109 patients with stable COPD and 95 control subjects, recruited at Clinical Department for Lung Diseases Jordanovac, University Hospital Centre Zagreb (Zagreb, Croatia). Complete blood count was performed on Sysmex XN-1000, CRP on Cobas c501, and Fbg on BCS XP analyser. Data were analysed with MedCalc statistical software.

**Results:**

Platelet (P = 0.007) and PLR (P = 0.006) were increased, while other platelet indices were decreased in COPD patients compared to controls. Combined model that included PLR, PDW and WBC showed great diagnostic performances, and correctly classified 75% of cases with an AUC of 0.845 (0.788 – 0.892), P < 0.001. Comorbidities (cardiovascular or metabolic diseases) had no effect on investigated parameters, while inhaled corticosteroids/long-acting β_2_-agonists (ICS/LABA) therapy increased MPV and PDW values in COPD patients.

**Conclusion:**

Platelet indices were altered in COPD patients and they could be valuable as diagnostic markers of COPD development, especially if combined with already known inflammatory markers.

## Introduction

Chronic obstructive pulmonary disease (COPD) is a complex and heterogeneous disease, additionally complicated by the presence of comorbidities, and is therefore often misdiagnosed and underestimated. However, in spite of that, it is projected to become the third leading cause of death by 2020. More than three million people worldwide die annually of COPD, making this disease an important public health problem. Cigarette smoking is considered the main COPD etiological factor. Still, only 15-20% smokers have COPD, and some other genetic (mostly yet unidentified, except for a_1_-antitrypsin deficiency) and non-genetic (air pollution and biomass fuel exposure, among others) factors also contribute significantly to COPD development (*1*). Main characteristic of the disease is chronic and irreversible airflow obstruction (*2*). Underlying mechanisms of COPD encompass protease-antiprotease imbalance, oxidative stress and chronic inflammation. It was shown that consequences of COPD are not present only locally (in pulmonary compartment), and systemic manifestations are present in most of COPD patients as well. Considering all of the abovementioned facts, some COPD endotypes and phenotypes have been proposed, yet many has to be discovered. According to precision medicine, patients within a certain subgroup should have a specific diagnostic and therapeutic approach (*3*). Today, therapeutic treatments are guided by the Global Initiative for Chronic Obstructive Lung Disease (GOLD) recommendations, and include short-acting β_2_-agonists (SABAs) or short-acting muscarinic antagonists (SAMAs) that improve forced expiratory volume in one second (FEV_1_) and COPD symptoms, long-acting β_2_-agonists (LABAs) and long-acting muscarinic antagonists (LAMAs) that improve lung function, or inhaled corticosteroids (ICS) that are often combined with long-acting bronchodilators (*1*).

It has been shown that FEV_1_ is poorly associated with the symptoms, health status, exercise capacity and other relevant characteristics of COPD (*4*). Therefore, various parameters that are increased or decreased in the circulation due to the disease could be important in understanding of its development and progression, response to therapy and effects of comorbidities (*5*). Systemic inflammation is recognized as a risk factor for many comorbidities in patients with COPD, which puts them at a greater risk of hospitalization and mortality (*6*). An increase in white blood cells (WBC) and a decrease in lymphocyte count was reported in COPD patients compared to healthy subjects as well as an increase in C-reactive protein (CRP), fibrinogen (Fbg), and inflammatory cytokines’ concentration (*4*, *6*–*8*). Inflammation was associated with the changes in structure, shape and dynamics of platelets (*9*). Platelet indices that describe mentioned changes are platelet count (Plt), mean platelet volume (MPV), platelet distribution width (PDW) and plateletcrit (Pct). Platelets recruit leukocytes to the site of inflammation and begin many intercellular and intracellular processes that may further take part in atherogenic and thrombotic events (*6*, *10*). Mean platelet volume and PDW are considered the markers for platelet activation that are commonly increased in patients with thrombotic and atherogenic risk (*6*, *11*). However, in COPD patients, beside an increase, a decrease in MPV was also reported due to the burden of inflammation presentable as exacerbation, intensive degradation of platelets or utilization of larger platelets at site of inflammation during the intercellular reactions (*7*, *10*, *12*, *13*). Platelet distribution width gives information about diversity of platelet size, so there is an increase in PDW in case of platelet hyperproduction, which cause the release of immature larger platelets from bone marrow (*14*). Plateletcrit is a parameter that describes the blood volume occupied by platelets. Regarding the definition of Pct and its formula, Pct = Plt x MPV / 10,000, it might be a kind of mechanism to maintain haemostasis by keeping the platelet mass unchanged (*13*). It was associated with various cardiovascular events, but also with COPD (*6*).

There is a need for information about changes in platelet indices in COPD patients. They are easily available in a daily laboratory routine, and if put in corresponding combinations they might achieve better predictive or diagnostic values. Thrombocytosis is often followed by lymphopenia, so if the counts of platelets and lymphocytes are calculated in platelet to lymphocyte ratio (PLR), it could become more reliable in describing the inflammation status. Indeed, PLR showed a very good performance as a potential inflammatory marker in various inflammatory diseases, including COPD (*6*, *15*). In addition, MPV/Plt ratio had a greater diagnostic value than MPV alone in pulmonary embolism, and it was a promising prognostic marker for lethal outcome in severe sepsis (*16*, *17*).

This study aimed to determine differences in platelet-related and common inflammatory parameters (CRP, Fbg, WBC) between control group and patients with stable COPD, according to the GOLD stages and ABCD clinical assessment. The hypothesis was that platelet indices are disturbed in COPD patients, and this would be accompanied by increased CRP, Fbg and WBC. Thus, in addition to CRP, Fbg and WBC, platelets as well as platelet-related parameters (MPV, PDW, Pct), and ratios (MPV/Plt, PDW/Plt, MPV/Pct and PDW/Pct) were investigated. Only PLR was explored in COPD so far. In addition, Plt index that unites Plt, Pct, MPV and PDW in one parameter was used, with a formula Plt index = MPV x PDW / Plt x Pct (*18*). Moreover, prognostic value of platelet indices was investigated. Finally, influence of several general comorbidities and commonly used inhalation therapies on platelet-related parameters was assessed, as data on these important topics are scarce.

## Materials and methods

### Subjects

Present study was retrospective and included a total of 204 individuals - 109 in a stable phase of COPD and 95 in control group. The study was approved by Ethical Committee of University Hospital Centre Zagreb (Zagreb, Croatia) and by Ethical Committee for Experimentation of Faculty of Pharmacy and Biochemistry, University of Zagreb (Zagreb, Croatia). All participants signed an informed consent for scientific research and agreed to take part in it as volunteers. It does not imply consent to publish personal individual data (names, pictures, hospital identification).

**Figure 1:**
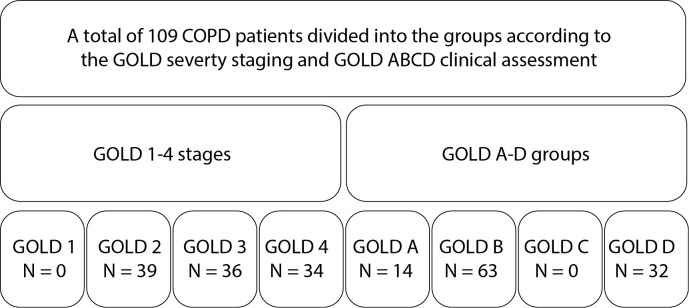
Subdivision of 109 COPD patients according to the severity of airflow limitation (GOLD 1-4 stages) and ABCD assessment (GOLD A-D groups). COPD – chronic obstructive pulmonary disease. GOLD - Global Initiative for Chronic Obstructive Lung Disease.

Patients with COPD were screened for eligibility and recruited during ambulatory visits at Clinical Department for Lung Diseases Jordanovac, University Hospital Centre Zagreb, during 2017 and 2018. Consecutive patients, mostly current or former smokers of tobacco, aged 65 (45–87) years, with objectively confirmed COPD according to the GOLD guidelines were included. Chronic obstructive pulmonary disease was diagnosed by a specialist pulmonologist based on anamnesis and clinical review, current symptoms and spirometry measurements. According to the GOLD criteria, COPD patients had FEV_1_/ forced vital capacity (FVC) value < 0.70, and they were subdivided into the groups based on airflow limitation grade (GOLD 1-4 stages) (*1*). Patients with FEV_1_ ≥ 80%, were in GOLD 1, with 50% ≤ FEV_1_< 80% were in GOLD 2, 30% ≤ FEV_1_ < 50% is a criterion for GOLD 3, and FEV_1_ < 30% for GOLD 4. Additionally, COPD patients were subdivided based on ABCD assessment using COPD Assessment Test (CAT) questionnaire (GOLD A-D groups) (Figure 1). All COPD patients included in the study had to be in stable phase of the disease, which was defined as no exacerbations during at least three visits in the previous 4 months, with no changes in respiratory medication and no symptoms of a lower respiratory tract infection. Apart from recent exacerbations and lung function parameters, other exclusion criteria for COPD patients were: age under 40, lung diseases other than COPD, inflammatory systemic diseases, acute infections, diabetes with severe complications, severe liver diseases, severe kidney insufficiency, malignant diseases, transplantations, and other specific or non-specific acute inflammations.

The control group was consisted of smokers and non-smokers, and their health state was established based on anamnestic data and spirometry test results. Control individuals had to meet the same inclusion and exclusion criteria as the COPD patients, except for the findings of post-bronchodilator spirometry test results (that were normal for control subjects).

### Methods

#### Laboratory tests

Determination of platelet indices as well as some general inflammatory biomarkers was evaluated in the blood samples, obtained by venepuncture of a large antecubital vein, of patients with stable COPD and controls.

All participants (patients and controls) were told in advance not to eat for (at least) 8 hours before the blood collection that was performed between 7 and 9 a.m. Fasting state was self-reported, and checked by nurse before blood collection.

For each individual, three blood tubes were drawn: for complete blood count (CBC) measurement – tube with K_3_EDTA anticoagulant for whole blood analysis (Greiner Bio-One, GmbH, Kremsmünster, Austria; volume 3 ml), for fibrinogen measurement – tube with 3.2% sodium citrate for plasma analysis (Becton, Dickinson and Company, Franklin Lakes, NJ, USA; volume 4.5 ml), and for CRP measurement – tube with gel without additive for serum analysis (Greiner Bio-One, GmbH, Kremsmünster, Austria; volume 5 ml). First, the blood was extracted into a coagulation tube with sodium citrate and mixed by an inversion of 3-4 times, then into a biochemical tube with gel and finally into a hematologic tube with K_3_EDTA (both tubes were mixed with an inversion of 8 times). For venepuncture and order of blood sampling and mixing, the guidelines were followed according to the national recommendations for venous blood sampling (*19*).

Complete blood count analysis was performed within half an hour after blood collection. Serum tube with gel was centrifuged at 2000xg for 10 min, as recommended by the manufacturer, and coagulation tube with sodium citrate was centrifuged two times at 1500xg for 15 min, as recommended by the Clinical and Laboratory Standards Institute (CLSI) guidelines, and by the manufacturer (*20*-*22*). Immediately thereafter, CRP and Fbg were measured.

Leukocyte, lymphocyte and Plt counts, as a part of CBC, were performed on Sysmex XN-1000 analyser (Sysmex Corporation, Kobe, Japan). Determination of leukocyte count and proportion of lymphocytes is based on the flow cytometry method where laser light scattering technology is used. Platelet counts and their respective indices are performed using the impedance method with hydrodynamic focusing. A platelet volume distribution curve is produced utilizing two thresholds: the lower move from 2 to 6 fL and the upper from 12 to 30 fL. The MPV was obtained by dividing Pct by Plt number. Platelet distribution width, a measure of platelet anisocytosis, is the width (measured as fL) of the size distribution curve at 20% of the peak. Immunoturbidimetry was a method used for the CRP determination on Cobas c501 analyser (Roche Diagnostics GmbH, Mannheim, Germany). The measurement of Fbg was performed on BCS XP analyser (Siemens Healthcare Diagnostics, Marburg, Germany).

Internal quality control and external quality assessment were performed for all parameters during the study period, according to HRN EN ISO 15189:2012 Medical laboratories - Requirements for quality and competence. The analysers were calibrated according to the manufacturer’s instructions and checked by using commercial controls. For CRP measurements commercial controls were used two times a day (PreciControl ClinChem Multi 1 (PCCC1; cat. no: 511 700 3190) and PreciControl ClinChem Multi 2 (PCCC2; cat. no: 511 721 6190), Roche Diagnostics GmbH, Mannheim, Germany) as well as for fibrinogen measurements (Control plasma N (cat. no: 05-ORKE41) and Control plasma P (cat. no: OUPZ175), Siemens Healthcare Diagnostics, Marburg, Germany). For CBC measurements commercial control (Sysmex XN Check Level 1 (cat. no: 213 487), Level 2 (cat. no: 213 488), Level 3 (cat. no: 213 489), Sysmex Corporation, Kobe, Japan) was used once a day.

#### Spirometry

Spirometry is a common method in diagnosing the airflow limitation. It enables to measure FVC, that is a volume of vigorously exhaled air from the point of maximal inspiration, and FEV_1_, the other important parameter that stands for the volume of air exhaled during the first second of previously described procedure for measuring FVC. Afterwards, FEV_1_/FVC is calculated. If its value is < 0.70, the airflow limitation is confirmed.

The spirometry was performed on each visit of COPD patients in outpatient clinic by trained technicians. The spirometry was done on a MasterScreen Pneumo (Jaeger, Germany), according to the recommendations of the European Respiratory Society and American Thoracic Society.

#### Comorbidities and therapy

Study participants provided a detailed medical history that included comorbidities and therapy data. Both COPD patients and controls were subdivided according to the presence of cardiovascular diseases (CVD) and metabolic diseases (MD). In this study, term CVD encompassed arterial hypertension, atherosclerosis, coronary artery disease and heart failure, while term MD encompassed diabetes mellitus, osteoporosis and hyperlipidaemia.

Furthermore, COPD patients were also assigned to the groups according to the therapy so that the effect of various therapies on platelets and platelet-related parameters could be determined. The patients were divided into four therapy groups, according to the GOLD guidelines based on medical data. Therapy 1 group was taking SABA, SAMA, LAMA or LABA and every possible dual combination among them. Long-acting β2-agonists and LAMA as a constant combination were used in therapy 2 group, while therapy 3 group was taking ICS/LABA combination. Finally, addition of LAMA to ICS/LABA combination was taken by the individuals in therapy 4 group.

### Statistical analysis

All data were tested for normal distribution by Kolmogorov-Smirnov test. As all data showed to be non-parametric, they were presented as median with interquartile range, and only age was presented as median with minimum and maximum. Accordingly, correlations were evaluated by Spearman Rank Order. Differences between controls and COPD groups were tested by Mann-Whitney Rank Sum Test, while in case of more than two groups Kruskal-Wallis One Way Analysis of Variance on Rank was used. Univariate and multivariate logistic regression analysis were performed for evaluation of predicting factors in COPD. Data were considered statistically significant when P < 0.05. Statistical analysis was performed by MedCalc statistical software, version 17.9.2. (MedCalc Software, Ostend, Belgium).

## Results

Table 1 shows baseline characteristics of all participants included (age, sex, smoking status, spirometric data) as well as results of laboratory testing. Controls and patients groups did not differ according to age (64 (46-83) and 65 (45-87), respectively) and sex (49 male and 46 female *vs.* 69 male and 40 female, respectively). All lung function parameters were significantly lower in subjects with COPD, as expected. Well-known inflammatory parameters, CRP, Fbg and WBC, showed increased levels in COPD patients compared to controls (P < 0.001 for all three parameters). An increase in Plt (P = 0.007), and a decrease in MPV (P < 0.001) and PDW (P < 0.001) compared to control subjects were found. Among the other platelet-related parameters, Pct for itself did not show significant change between COPD patients and controls (P = 0.220), but when combined with MPV or PDW in a MPV/Pct or PDW/Pct, lower result in COPD patients were obtained (P = 0.005 and P < 0.001, respectively). The same principle of calculation was used on Plt, so the MPV/Plt and PDW/Plt ratios were better in distinguishing COPD patients and controls (P < 0.001, P < 0.001, respectively) than Plt itself. Platelet index was decreased in COPD patients when compared to control subjects (P < 0.001).

**Table 1:** Baseline characteristics, spirometric, inflammatory and platelet-related parameters of controls and patients with stable COPD

**Parameter**	**Controls** **N = 95**	**COPD** **N = 109**	**P-value**
**age (years)**	64 (46-83)	65 (45-87)	0.069
**sex**			
**males; N/total**	49/95	69/109	0.121
**females; N/total**	46/95	40/109	
**smoking status**			
**current smokers, N (%)**	47 (49%)	29 (27%)	
**former smokers, N (%)**	0 (0%)	75 (69%)	< 0.001
**never smokers, N (%)**	48 (51%)	5 (4%)	
**FEV_1_ (L)**	2.60 (2.12-3.19)	1.08 (0.69-1.60)	< 0.001
**FEV_1_ (%)**	93 (86-104)	41 (28-62)	< 0.001
**FVC (L)**	3.35 (2.77-4.16)	2.28 (1.74-2.77)	< 0.001
**FEV_1_/FVC**	0.81 (0.77-0.88)	0.51 (0.41-0.59)	< 0.001
**CRP (mg/L)**	1.47 (0.74-2.78)	2.34 (1.15-4.67)	< 0.001
**Fbg (g/L)**	3.5 (3.1-3.8)	3.8 (3.4-4.5)	< 0.001
**WBC (x10^9^/L)**	6.14 (5.15-7.42)	7.57 (6.56-8.95)	< 0.001
**Plt (x10^9^/L)**	219 (198-260)	241 (215-278)	0.007
**lymphocytes (x10^9^/L)**	1.93 (1.64-2.40)	1.92 (1.50-2.32)	0.243
**PLR**	122 (90-139)	132 (102-163)	0.006
**MPV (fL)**	10.6 (9.9-11.1)	10.0 (9.5-10.6)	< 0.001
**PDW (%)**	12.8 (11.8-14.2)	11.1 (10.2-12.8)	< 0.001
**MPV/Plt**	0.047 (0.040-0.055)	0.041 (0.036-0.047)	< 0.001
**PDW/Plt**	0.057 (0.047-0.068)	0.046 (0.039-0.056)	< 0.001
**Pct**	0.002 (0.002-0.003)	0.002 (0.002-0.003)	0.220
**MPV/Pct**	4565 (3844-5104)	4125 (3586 - 4667)	0.005
**PDW/Pct**	5478 (4656-6180)	4621 (4037-5371)	< 0.001
**Plt index**	259 (189-341)	190 (147-251)	< 0.001
Smoking status is presented as absolute numbers (percentages), and all other data are presented as the median (interquartile range), except for age that is presented as median (minimum – maximum); data were analysed by Mann-Whitney Rank Sum test. COPD – chronic obstructive pulmonary disease. FEV_1_ - forced expiratory volume in 1 second. FVC - forced vital capacity. CRP - C-reactive protein. Fbg – fibrinogen. WBC - white blood cells. Plt - platelet count. PLR - platelet to lymphocyte ratio. MPV - mean platelet volume. PDW - platelet distribution width. Pct - plateletcrit.			

**Figure 2:**
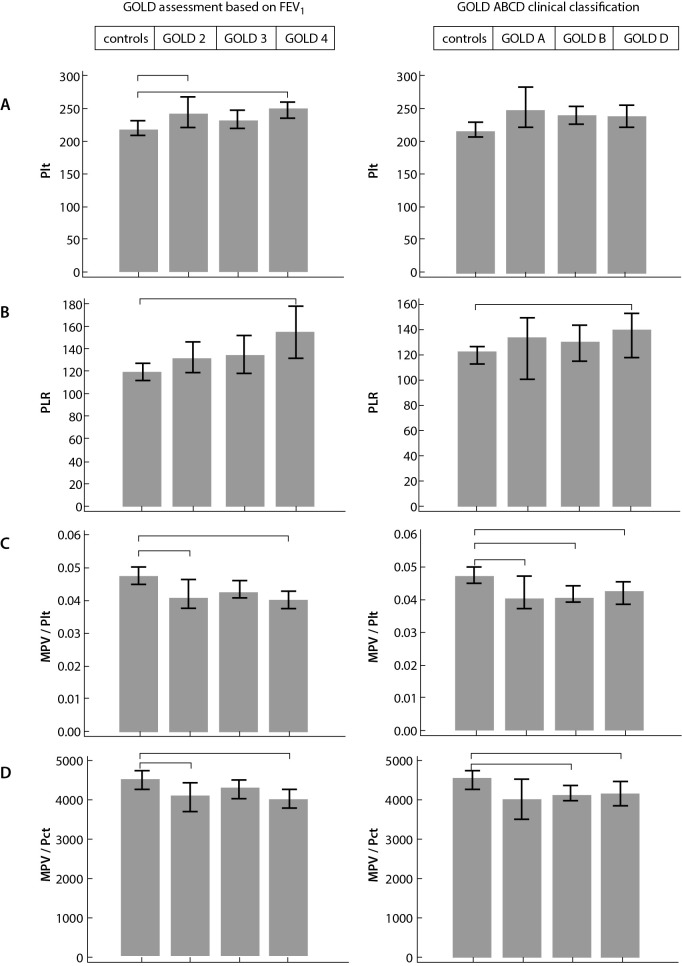
Values of Plt (A), PLR (B), MPV/Plt (C) and MPV/Pct (D) in control group and COPD groups subdivided by the severity of airflow limitation (GOLD 2-4 stages) and ABCD assessment (GOLD A-D groups). Results are shown as the median (interquartile range). GOLD 2-4 stages and GOLD A-D groups, compared to total number of controls included in the study, were tested by Kruskal-Wallis One Way Analysis of Variance on Ranks. Connectors on the graphs show between which groups was observed statistically significant difference with P < 0.05. GOLD - Global Initiative for Chronic Obstructive Lung Disease. Plt - platelet count. PLR - platelet to lymphocyte ratio. MPV - mean platelet volume. PDW - platelet distribution width. Pct - plateletcrit.

Platelet to lymphocyte ratio was significantly elevated in COPD patients (P = 0.006). Moreover, PLR showed to be related to the disease severity, as a statistically significant differences were found between controls and GOLD 4 (P < 0.05) as well as between controls and GOLD D (P < 0.05). Platelet to lymphocyte ratio was not the only parameter that showed the change among GOLD 2-4 stages and GOLD A-D subgroups. Platelets also showed changes when patients were subdivided into GOLD 2-4 stages, but there was no significant change between GOLD A-D groups when compared to controls and between individual groups. MPV/Plt and MPV/Pct ratios showed a downtrend with statistical significance between different groups, as shown in Figure 2.

Fibrinogen demonstrated a poor positive correlation with Plt (r = 0.40, P < 0.001) and Pct (r = 0.43, P < 0.001) in COPD patients. Negative correlation was observed with MPV/Plt (r = - 0.32, P < 0.001), MPV/Pct (r = - 0.40, P < 0.001), PDW/Plt (r = - 0.35, P < 0.001), PDW/Pct (r = - 0.29, P = 0.002) and Plt index (r = - 0.35, P < 0.001). Among all of the mentioned platelet-related parameters in control group, Fbg showed a negative correlation with PLR only (r = - 0.37, P < 0.001). In addition, univariate logistic regression analysis (Table 2) showed that Fbg has the highest odds ratio value of all individual parameters tested (OR = 2.55 (95% CI = 1.65-3.95), P < 0.001). Next, for multivariate logistic regression analysis all the parameters that showed statistically significant results in univariate logistic regression were included. As it is shown in Table 3. WBC, PDW and PLR gave the best combination for COPD prediction with its area under the curve (AUC) of 0.845 (95% CI = 0.788–0.892, P < 0.001) and 75% correctly classified cases.

**Table 2:** Univariate logistic regression analysis with given predictors and protectors for COPD

**Parameter**	**Odds ratio**	**95% CI**	**P-value**	**Correctly classified cases (%)**
**CRP**	1.24	1.09-1.41	< 0.001	58.8
**Fbg**	2.55	1.65-3.95	< 0.001	60.8
**WBC**	1.48	1.25-1.76	< 0.001	51.0
**Plt**	1.01	1.00-1.01	0.015	61.8
**PLR**	1.01	1.00-1.02	0.004	54.4
**MPV**	0.59	0.43-0.80	< 0.001	62.8
**PDW**	0.72	0.62-0.84	< 0.001	63.7
**PDW/Pct**	1.00	1.00-1.00	0.003	62.7
CI – confidence interval. CRP - C-reactive protein. Fbg – fibrinogen. WBC - white blood cells. Plt – platelet. PLR - platelet to lymphocyte ratio. MPV - mean platelet volume. PDW - platelet distribution width. Pct - plateletcrit.				

**Table 3:** Multivariate logistic regression analysis of parameters whose results were statistically significant in univariate logistic regression analysis

**parameter**	**odds ratio**	**95% CI**	**P-value**
**PLR**	1.02	1.01-1.03	< 0.001
**PDW**	0.55	0.42-0.72	< 0.001
**WBC**	1.89	1.43-2.51	< 0.001
The analysis gave results with 75% correctly classified cases and area under curve of 0.845 (0.788–0.892). Only statistically significant results are shown in the table. CI – confidence interval. PLR - platelet to lymphocyte ratio. PDW - platelet distribution width. WBC - white blood cells.			

Furthermore, controls and patients were subdivided into the groups depending on the presence of observed comorbidities (CVD or MD), and information about the levels of platelets and their indices was provided. There were no statistically significant changes between COPD patients with comorbidity and COPD patients without comorbidity. In cases of statistically relevant results, they were observed between control groups when compared to COPD groups with or without comorbidity (Table 4).

**Table 4:** Effect of various comorbidities on platelets and platelet-related parameters

**Parameter**	**Comorbidity**	**Controls 1**	**Controls 2**	**COPD 1**	**COPD 2**	**P-value**
**Plt (x10^9^/L)**	CVD	212 (192-264)	224 (199-259)	251 (218-285)	239 (214-257)	0.044^a,b^
	MD	228 (198-268)	217 (196 - 248)	247 (220-278)	239 (212-277)	0.031^b,d^
**PLR**	CVD	119 (92-137)	121 (90-139)	136 (103-159)	133 (101-171)	0.050
	MD	125 (111-143)	119 (90-137)	123 (105-169)	136 (101-161)	0.022^b,d^
**MPV (fL)**	CVD	10.5 (10.1-11.4)	10.7 (9.9-11.1)	10.0 (9.4-10.6)	10.0 (9.5-10.6)	0.001^a,b,c,d^
	MD	10.7 (9.6-11.1)	10.6 (10.0-11.1)	9.8 (9.3-10.5)	10.1 (9.6-10.7)	< 0.001^a,b,d^
**PDW (%)**	CVD	12.8 (12.0-14.3)	12.9 (11.7-14.2)	11.1 (10.2-12.6)	11.2 (10.2-12.8)	< 0.001^a,b,c,d^
	MD	12.7 (11.3-14.2)	12.9 (11.9-14.2)	10.8 (10.0-12.1)	11.3 (10.4-13.0)	< 0.001^a,b,c,d^
**Pct**	CVD	0.002 (0.002-0.003)	0.002 (0.002-0.003)	0.002 (0.002-0.003)	0.002 (0.002-0.003)	0.445
	MD	0.0025 (0.0021 - 0.0028)	0.0023 (0.0021 - 0.0026)	0.0024 (0.0022 - 0.0029)	0.0024 (0.0021 - 0.0028)	0.476
**Plt index**	CVD	282 (177-403)	257 (190- 325)	179 (134-262)	197 (162-247)	0.001^a,b,c,d^
	MD	250 (150-332)	268 (196-359)	185 (137-245)	194 (150-255)	0.001^b,d^
Results are presented as the median (interquartile range). Controls and chronic obstructive pulmonary disease (COPD) patients were subdivided based on the presence of comorbidity and they were tested by Kruskal-Wallis One Way Analysis of Variance on Ranks. controls 1 - controls with comorbidity. controls 2 - controls without comorbidity. COPD 1 - COPD patients with comorbidity. COPD 2 - COPD patients without comorbidity. CVD - cardiovascular diseases. MD - metabolic disorders. Plt - platelet count. PLR - platelet to lymphocyte ratio. MPV - mean platelet volume. PDW - platelet distribution width. Pct - plateletcrit. CVD - controls 1, N = 24. controls 2, N = 71. COPD 1, N = 56. COPD 2, N = 53. MD - controls 1, N = 25. controls 2, N = 70. COPD 1, N = 39. COPD 2, N = 70. ^a^statistically significant difference between controls 1 and COPD 1. ^b^statistically significant difference between controls 2 and COPD 1. ^c^statistically significant difference between controls 1 and COPD 2. ^d^statistically significant difference between controls 2 and COPD 2.						

Based on commonly used therapy - inhaled bronchodilators, either individually or in a combination, and ICS, COPD patients were subdivided into the groups according to if they were taking a specific therapy or not. The results regarding the influence of a specific therapy on platelets and platelet-related parameters are shown in Table 5. There were two statistically significant results, the level of MPV and the level of PDW were increased in COPD patients with therapy 3 (combination of LABA and ICS) when compared to COPD patients without therapy 3 (P = 0.038 and P = 0.026, respectively).

**Table 5:** Effect of chronic inhalation therapy on platelets and platelet-related parameters

**Parameter**	**Therapy type**	**With therapy**	**Without therapy**	**P-value**
	therapy 1	240 (210-269)	242 (215-280)	0.903
**Plt** **(x10^9^/L)**	therapy 2	234 (213-253)	242 (219-284)	0.268
	therapy 3	244 (223-295)	241 (214-277)	0.644
	therapy 4	243 (214-289)	239 (217-275)	0.756
	therapy 1	122 (110-143)	136 (101-171)	0.496
**PLR**	therapy 2	130 (103-154)	139 (101-169)	0.387
	therapy 3	138 (102-173)	133 (102-161)	0.764
	therapy 4	150 (96-173)	130 (103-151)	0.282
	therapy 1	9.8 (9.5-10.2)	10.0 (9.5-10.8)	0.294
**MPV (fL)**	therapy 2	9.9 (9.5-10.8)	10.0 (9.5-10.6)	0.894
	therapy 3	10.4 (10.0-11.2)	9.9 (9.4-10.5)	0.038
	therapy 4	10.0 (9.2-10.6)	9.9 (9.6-10.7)	0.456
	therapy 1	10.6 (10.3-11.7)	11.4 (10.2-12.9)	0.415
**PDW (%)**	therapy 2	11.2 (10.2-12.5)	11.1 (10.3-12.8)	0.910
	therapy 3	12.3 (11.1-14.0)	11.1 (10.1-12.3)	0.026
	therapy 4	11.3 (9.6-12.6)	11.1 (10.3-12.8)	0.387
	therapy 1	0.002 (0.002-0.003)	0.002 (0.002-0.003)	0.775
**Pct**	therapy 2	0.002 (0.002-0.003)	0.003 (0.002-0.003)	0.342
	therapy 3	0.003 (0.002-0.003)	0.002 (0.002-0.003)	0.166
	therapy 4	0.003 (0.002-0.003)	0.002 (0.002-0.003)	0.850
	therapy 1	182 (157-230)	193 (145-255)	0.627
**Plt index**	therapy 2	197 (157-266)	188 (134-245)	0.376
	therapy 3	218 (138-242)	188 (147-255)	0.590
	therapy 4	183 (134-260)	194 (152-248)	0.595
Results are presented as the median (interquartile range). COPD patients were subdivided based on the chronic inhalation therapy 1, 2, 3 or 4, and they were tested by Mann-Whitney test. therapy 1 - SABA or SAMA or LAMA or LABA. therapy 2 - LABA/LAMA. therapy 3 - ICS/LABA. therapy 4 - ICS/LABA+LAMA. SABA - short-acting β_2_-agonist. SAMA - short-acting muscarinic antagonist. LABA - long-acting β_2_-agonist. LAMA - long-acting muscarinic antagonist. ICS - inhaled corticosteroids. therapy 1: with, N = 20; without, N = 89. therapy 2: with, N = 32; without, N = 77. therapy 3: with, N = 16; without, N = 93. therapy 4: with, N = 37; without, N = 72. Plt - platelet count. PLR - platelet to lymphocyte ratio. MPV - mean platelet volume. PDW - platelet distribution width. Pct - plateletcrit.				

## Discussion

Study demonstrated that platelet indices were disturbed in COPD patients. Indeed, Plt and PLR were increased, while other platelet-related parameters were decreased in COPD patients group compared to control subjects. Combined model that included PLR, PDW and WBC showed great diagnostic performances, and correctly classified 75% of cases with an AUC of 0.845 (95% CI = 0.788–0.892). Chronic obstructive pulmonary disease patients with or without comorbidities (CVD or MD) had similar values for all investigated parameters, while ICS/LABA therapy increased MPV and PDW values in COPD patients.

Increased Plt in patients with stable COPD compared to age- and sex-matched controls, observed in this study, can be explained with the fact that an underlying inflammation in COPD stimulates bone marrow to synthesize platelets (*9*). In case of reduced platelet production, the newly synthesized platelets have larger volume and are more active, as a result of compensation (*16*, *17*, *23*). Increased MPV could be considered as a marker of platelet activation (*6*, *11*, *14*, *16*). However, there are some differences in results between the studies. In this study, MPV was decreased in patients with stable COPD in comparison to controls, which may suggest that platelets in the circulation are less active and, therefore, patients with stable COPD are at lower risk for thrombotic events. Some studies suggested that a decrease in MPV levels could be a consequence of involvement and utilization of larger platelets at site of inflammation. Therefore, smaller platelets remain and cause a reduction in MPV levels (*24*, *25*). Chronic obstructive pulmonary disease patients had lower MPV during an exacerbation episode, and the level increased after the recovery period (*12*). Accordingly, following up the MPV levels could be used for monitoring the recovery after the exacerbation (*7*). Beside a lower MPV in acute exacerbation, a decrease in MPV was also found before in stable phase of COPD when compared to control group (*12*). Therefore, it would be valuable to assess MPV in larger groups of controls and stable COPD patients as well as in COPD patients with exacerbations to clarify more controversial results obtained in some publications.

Mean platelet volume levels could differ in the studies as the consequence of preanalytical factors as much as the analysis itself (*25*). Indeed, factors such as age, gender, race, ethnicity, lifestyle and genetic background, the venepuncture method, the anticoagulant used, type or sample, and many more, as much as the analysis itself regarding a diversity of methods, could have an effect on MPV values (*25*). Moreover, inter-individual differences in response to the changes in the diseases should be considered as a factor that affects not only MPV (*9*, *11*). Besides MPV, the other marker used for detection of platelet activation is PDW, a parameter that describes platelet volume heterogeneity (*14*). Similar to MPV, PDW was also decreased in this study, which potentiated the suggestion on lower platelets’ activity in patients with stable COPD. Both MPV and PDW showed odds ratios lower than one in univariate logistic regression analysis (0.59 and 0.72, respectively), which means that they showed a protective role. Moreover, when combined with PLR and WBC in a multivariate logistic regression analysis, PDW showed even lower odds ratio of 0.55.

Some studies applied combinations of MPV and PDW with Plt and Pct (MPV/Plt, MPV/Pct, PDW/Plt, PDW/Pct, Plt index) when investigating different diseases and conditions, in an attempt to increase diagnostic performances of the ratios compared to individual parameters (*2*, *6*, *18*, *26*). Only PLR was investigated in COPD so far (*2*, *6*, *15*). In this study, from all individual parameters examined, only Plt distinguished COPD patients from controls regarding criteria for GOLD 2-4 stages, but not for GOLD A-D groups. However, even three different platelet-related ratios could provide distinction in comparison of either GOLD 2-4 stages or GOLD A-D groups with control subjects, namely MPV/Plt, MPV/Pct and PLR.

This investigation showed that PLR is increased in stable COPD, with statistically different results being observed between control group and GOLD 4 as well as between control group and GOLD D. Platelet to lymphocyte ratio can be used as a diagnostic indicator of systemic inflammation. It is better parameter than Plt and lymphocyte count individually because it associates their general inverse relation and reduces the impact of preanalytical factors, such as blood specimen handling and level of hydration, on individual parameters (*6*, *27*). Moreover, when PLR, PDW and WBC were combined, this three-component model showed a very good diagnostic prediction for the presence of COPD (75% of cases could be correctly classified). Diagnostic performances of this combination exceeded those of any individual platelet-related or common inflammatory parameters that were also explored in this study.

Patients with stable COPD had increased levels of inflammatory proteins (CRP and Fbg) and cells (WBC), which are non-invasive, easily measured, inexpensive parameters (*28*). Elevated Fbg was more than two times expected to be seen in patients with stable COPD than in controls (OR = 2.55). According to that, Fbg seems to be a good COPD predictor; however, this interpretation should be taken with caution, as Fbg is a non-specific acute phase parameter. Moreover, Fbg positively (Plt, Pct) or negatively (MPV/Plt, MPV/Pct, PDW/Plt, PDW/Pct, Plt index) correlated with platelet indices in COPD patients.

It was shown that increased Fbg is associated with reduced lung function and increased risk of COPD (*29*). Many COPD patients have cardiovascular comorbidities, and Fbg is also associated with cardiovascular mortality (*28*). However, in this study no significant differences in Fbg values were found between individuals (controls or COPD patients) with CVD and those without CVD (data not shown).

Regarding platelets and their related parameters, statistically relevant results were not observed either between control individuals with and without comorbidities or between COPD patients with and without comorbidities (CVD or MD). Further studies with larger subgroups having those or some other common comorbidity are needed for results to be more significant.

In order to improve the quality of life (by affecting lung function and/or underlying inflammation), COPD patients are subjected to long-lasting therapy regimes that are adjusted to their current symptoms and overall state. Patients in this study received four different chronic inhalation treatments. Those therapy regimes affected MPV and PDW only; specifically, ICS/LABA combination increased their values. Data of influence on COPD therapy on haematological or biochemical blood parameters are rarely found. It is necessary to do more research with the aim to determine if the reason for no changes is in inefficient therapy or platelet-related parameters do not have a potential in monitoring the therapy (*30*).

There are several shortcomings of this study. The sample size was relatively small, especially for subgroups with respect to the disease severity (either GOLD 2-4 stages or GOLD A-D groups), the presence of comorbidity and therapy. In addition, no patient with GOLD 1 stage participated in this study and it would be interesting to investigate the pattern of platelet indices at the beginning of the disease development; however, unfortunately, this group of COPD patients rarely contact their physician due to very mild symptoms. At the beginning, patients were recruited according to the inclusion criteria and later on were subdivided according to the GOLD category A-D. This is the reason why no patient in the GOLD C category participated in this study. However, these results are consistent with available data showing that GOLD C category of patients is very rare (patients that does not have many symptoms usually are not frequent exacerbators).

It was observed that MPV and PDW have a potential of being protective parameters and that could be evaluated if a group of COPD patients with exacerbations is included in the study. According to that, large-scale prospective study for evaluation and validation of the results from current study should be considered.

In conclusion, after determining potentially good COPD biomarkers, it is required to assess their reproducibility for clinical practice. With a combination of several different biomarkers, better clinical protocol in COPD diagnostics, therapy monitoring and defining of new phenotypes and endotypes could be achieved. When combining PLR, PDW and WBC, 75% of cases were correctly classified in this study, and a very good diagnostic power between controls and COPD subjects was accomplished. Considering many existing changes in thrombotic events, platelets could become an attractive diagnostic and therapeutic target. Another important advantage is that platelets and their indices are common, inexpensive and non-invasive tests performed routinely in everyday laboratory practice.

## References

[1] GOLD commitee. Global Initiative for Chronic Obstructive Lung Disease (GOLD 2019). 2019;2–14. Available at: www.goldcopd.org. Accessed February 15th 2019.

[2] Yao C Liu X Tang Z Prognostic role of neutrophil–lymphocyte ratio and platelet–lymphocyte ratio for hospital mortality in patients with AECOPD. Int J Chron Obstruct Pulmon Dis. 2017;12:2285–90. 10.2147/COPD.S141760 28814856 PMC5546734

[3] Sidhaye VK Nishida K Martinez FJ Precision medicine in COPD: where are we and where do we need to go? Eur Respir Rev. 2018;27:180022. 10.1183/16000617.0022-2018 30068688 PMC6156790

[4] Agusti A Sin DD Biomarkers in COPD. Clin Chest Med. 2014;35:131–41. 10.1016/j.ccm.2013.09.006 24507841

[5] Barnes PJ Inflammatory mechanisms in patients with chronic obstructive pulmonary disease. J Allergy Clin Immunol. 2016;138:16–27. 10.1016/j.jaci.2016.05.011 27373322

[6] Kalemci S Akin F Sarihan A Sahin C Zeybek A Yilmaz N Relationship between hematological parameters and severity of chronic obstructive pulmonary disease. Pol Arch Intern Med. 2018;128:171–7. 10.20452/pamw.4198 29385111

[7] Koç I Karataş ZA Mandollu E Mermer A Kaya A Dokme A Importance of mean platelet volume in patients with chronic obstructive pulmonary disease. Gaziantep Med J. 2014;20:294–8. 10.5455/GMJ-30-154104

[8] Kim TH Oh DK Oh YM Lee SW Do Lee S Lee JS Fibrinogen as a potential biomarker for clinical phenotype in patients with chronic obstructive pulmonary disease. J Thorac Dis. 2018;10:5260–8. 10.21037/jtd.2018.08.52 30416773 PMC6196189

[9] Klinger MHF Jelkmann W Review: Role of Blood Platelets in Infection and Inflammation. J Interferon Cytokine Res. 2002;22:913–22. 10.1089/10799900260286623 12396713

[10] Biljak VR Pancirov D Cepelak I Popović-Grle S Stjepanović G Grubišić TŽ Platelet count, mean platelet volume and smoking status in stable chronic obstructive pulmonary disease. Platelets. 2011;22:466–70. 10.3109/09537104.2011.573887 21506665

[11] Malerba M Clini E Malagola M Avanzi GC Platelet activation as a novel mechanism of atherothrombotic risk in chronic obstructive pulmonary disease. Expert Rev Hematol. 2013;6:475–83. 10.1586/17474086.2013.814835 23991933

[12] Wang RT Li JY Cao ZG Li Y Mean platelet volume is decreased during an acute exacerbation of chronic obstructive pulmonary disease. Respirology. 2013;18:1244–8. 10.1111/resp.12143 23786593

[13] Budak YU Polat M Huysal K The use of platelet indices, plateletcrit, mean platelet volume and platelet distribution width in emergency non-traumatic abdominal surgery: a systematic review. Biochem Med (Zagreb). 2016;26:178–93. 10.11613/BM.2016.020 27346963 PMC4910273

[14] Wang M Zhang J Ji Q Yang Q Zhao F Li W Evaluation of platelet distribution width in chronic obstructive pulmonary disease patients with pulmonary embolism. Biomark Med. 2016;10:587–96. 10.2217/bmm.15.112 26567584

[15] Kumar P Law S Sriram KB Evaluation of platelet lymphocyte ratio and 90-day mortality in patients with acute exacerbation of chronic obstructive pulmonary disease. J Thorac Dis. 2017;9:1509–16. 10.21037/jtd.2017.05.77 28740663 PMC5506124

[16] Oh GH Chung SP Park YS Hong JH Lee HS Chung HS Mean platelet volume to platelet count ratio as a promising predictor of early mortality in severe sepsis. Shock. 2017;47:323–30. 10.1097/SHK.0000000000000718 27504801

[17] Yardan T Meric M Kati C Celenk Y Atici AG Mean platelet volume and mean platelet volume/platelet count ratio in risk stratification of pulmonary embolism. Medicina (Kaunas). 2016;52:110–5. 10.1016/j.medici.2016.03.001 27170484

[18] Golwala ZM Shah H Gupta N Sreenivas V Puliyel JM Mean platelet volume (MPV), platelet distribution width (PDW), platelet Count and plateletcrit (PCT) as predictors of in-hospital paediatric mortality: A case-control study. Afr Health Sci. 2016;16:356–62. 10.4314/ahs.v16i2.3 27605950 PMC4994558

[19] Nikolac N Supak-Smolcić V Simundić AM Celap I Croatian Society of Medical Biochemistry and Laboratory Medicine: National recommendations for venous blood sampling. Biochem Med (Zagreb). 2013;23:242–54. 10.11613/BM.2013.031 24266294 PMC3900082

[20] Greiner Bio-One. VACUETTE Blood Collection System Handling Recommendations. Available at: https://www.gbo.com/fileadmin/user_upload/Downloads/Brochures/Brochures_Preanalytics/English/980102_Handhabungsempfehlungen_rev09_0314_e_lowres.pdf. Accessed February 15th 2019.

[21] Clinical and Laboratory Standards Institute (CLSI). Collection, Transport, and Processing of Blood Specimens for Testing Plasma-Based Coagulation Assays and Molecular Hemostasis Assays – Fifth Edition. CLSI Document H21-A5. Wayne, PA, 2008.

[22] BD Life Sciences. Preanalytical Systems BD Life Sciences Product Catalogue. Available at: https://www.bd.com/resource.aspx?IDX=34369. Accessed February 15th 2019.

[23] Mukker P Kiran S Platelet indices evaluation in patients with dengue fever. Int J Res Med Sci. 2018;6:2054–9. 10.18203/2320-6012.ijrms20182287

[24] Koçak MZ Analysis of mean platelet volume in chronic obstructive pulmonary disease patients during acute attack. Biomed Res (Aligarh). 2017;28:2783–5.

[25] Korniluk A Koper-Lenkiewicz OM Kamińska J Kemona H Dymicka-Piekarska V Mean Platelet Volume (MPV): New Perspectives for an Old Marker in the Course and Prognosis of Inflammatory Conditions. Mediators Inflamm. 2019;2019: 9213074. 10.1155/2019/9213074 31148950 PMC6501263

[26] Elsayed AM Mohamed GA Mean platelet volume and mean platelet volume/platelet count ratio as a risk stratification tool in the assessment of severity of acute ischemic stroke. Alexandria J Med. 2017;53:67–70. 10.1016/j.ajme.2016.03.003

[27] Kurtipek E Bekci TT Kesli R Erdem SS Terzi Y The role of neutrophil-lymphocyte ratio and platelet-lymphocyte ratio in exacerbation of chronic obstructive pulmonary disease. J Pak Med Assoc. 2015;65:1283–7. 26627508

[28] Duvoix A Dickens J Haq I Mannino D Miller B Tal-Singer R Blood fibrinogen as a biomarker of chronic obstructive pulmonary disease. Thorax. 2013;68:670–6. 10.1136/thoraxjnl-2012-201871 22744884 PMC3711372

[29] Dahl M Tybjaerg-Hansen A Vestbo J Lance P Nordestgaard BG Elevated plasma fibrinogen associated with reduced pulmonary function and increased risk of chronic obstructive pulmonary disease. Am J Respir Crit Care Med. 2001;164:1008–11. 10.1164/ajrccm.164.6.2010067 11587987

[30] Ekström MP Hermansson AB Ström KE Effects of cardiovascular drugs on mortality in severe chronic obstructive pulmonary disease: A time-dependent analysis. Am J Respir Crit Care Med. 2013;187:715–20. 10.1164/rccm.201208-1565OC 23328521

